# Higher Total Cerebral Small Vessel Disease Burden Was Associated With Mild Cognitive Impairment and Overall Cognitive Dysfunction: A Propensity Score-Matched Case–Control Study

**DOI:** 10.3389/fnagi.2021.695732

**Published:** 2021-07-12

**Authors:** Xuanting Li, Junliang Yuan, Wei Qin, Lei Yang, Shuna Yang, Yue Li, Wenli Hu

**Affiliations:** ^1^Department of Neurology, Beijing Chaoyang Hospital, Capital Medical University, Beijing, China; ^2^NHC Key Laboratory of Mental Health, Peking University, Beijing, China; ^3^National Clinical Research Center for Mental Disorders, Peking University Sixth Hospital, Beijing, China; ^4^Department of Neurology, Peking University Sixth Hospital, Peking University Institute of Mental Health, Beijing, China

**Keywords:** cerebral small vessel disease, mild cognitive impairment, propensity score matching, susceptibility weighted imaging, neuropsychology

## Abstract

**Background and Objective:**

The combination of neuroimaging and cognition characteristics may provide complementary information for early identification of mild cognitive impairment (MCI). This study aimed to establish the clinical relevance between cerebral small vessel disease (CSVD) burden and MCI and further explored the cognitive characteristics linked to CSVD applying a propensity score matching (PSM) approach.

**Methods:**

The study was designed as a case–control study. All the subjects underwent the standard clinical assessments, neuropsychological testing battery (including global cognition, memory, executive function, and speed and motor control domains), and brain magnetic resonance imaging (MRI). A 1:2 nearest-neighbor matching approach without replacement was employed with a caliper of 0.15 in the PSM approach.

**Results:**

A total of 84 MCI patients and 186 cognitively normal controls were included in this study. After PSM, 74 MCI patients and 129 controls were successfully matched, and the covariate imbalance was well eliminated. Compared with controls, the MCI group had more severe CSVD burden. In the binary logistic regression analysis, CSVD was associated with MCI after adjusting for all confounders. The results of multivariate linear regression analyses showed that higher total MRI CSVD burden was related to the deficit of cognitive performance in global cognition and three important cognitive domains after adjusting for all confounders.

**Conclusion:**

Cerebral small vessel disease was an independent risk factor of MCI. Moreover, higher total MRI CSVD burden was associated with the overall cognitive impairment among middle-aged and elderly Chinese adults.

## Introduction

Mild cognitive impairment (MCI) refers to a special state between normal cognition and dementia, which is of great significance in understanding the process of cognitive decline. It has become a key point of clinical and intervention trials. MCI has a few different clinical features and subtypes, and the prevalence and prognosis vary depending on different etiologies (Tangalos and Petersen, 2018).

It is hypothesized that multiple-domain MCI, either amnestic or not, was considered to have a vascular etiology. Cerebral small vessel disease (CSVD), an important component of systemic vascular disease, has been found to have a close association with cognitive impairment (Salvadori et al., 2016). Most of prior studies were focused on the impact of a single imaging marker of CSVD on cognitive function, such as lacunar infarction, white matter hyperintensity (WMH), and cerebral microbleed (CMB). However, different CSVD markers subtend different pathophysiological processes. CSVD is currently considered to be a global and dynamically changing disease (Shi and Wardlaw, 2016; Ter Telgte et al., 2018). Therefore, it is necessary to clarify the effect of total CSVD burden on cognitive dysfunction and the characteristics of different cognitive domains in MCI.

Recently, studies have paid more attention to the clinical features of total CSVD burden shown on magnetic resonance imaging (MRI). A study from the Netherlands tested a new method to evaluate total brain damage in CSVD patients and found that accumulating brain damage was related to worse cognitive performance, especially in information processing speed and overall cognition (Huijts et al., 2013). A cross-sectional study found that global network efficiency mediated the relationship between total MRI CSVD burden and information processing speed (Heinen et al., 2018). Another cross-sectional study also found that the total CSVD burden had the clinical relevance in a memory clinic population and associated with the performance of frontal and visuospatial tasks (Banerjee et al., 2018).

A recent proposal of MCI diagnosis highlighted the need of an objective evidence in several cognitive domains (Sachdev et al., 2014). There remains controversy for the criteria and the operationalization of MCI related to CSVD because of the no real agreement about the assessed cognitive domains and the proper cut scores. Therefore, more studies are needed to prove the causal relationship between CSVD and MCI and to find out the characteristics in different cognitive domains among CSVD patients.

For an observational study, where there are likely confounders impacting both cognition and CSVD, the correlation of CSVD and MCI could be distorted by the imbalance of disease characteristics. Propensity score matching (PSM) is a useful method used to correct biased estimation of outcome differences in statistical analyses. This method allows researchers to estimate the causal relationship using observational or non-randomized data.

Due to the complexity of pathologic changes and clinical symptoms, the combination of non-invasive MRI and cognition characteristics may provide complementary information for early identification of MCI. Thus, our aim was to establish the clinical relevance between the CSVD MRI markers and MCI and further explore the cognitive features linked to CSVD among middle-aged and elderly Chinese, applying the PSM approach to balance covariates between MCI patients and controls. We hypothesized that total MRI CSVD burden might be an important cause of MCI and would be particularly associated with “vascular” domains such as executive function and speed and motor control.

## Materials and Methods

### Subjects

This was a case–control study designed to evaluate the influence of total MRI CSVD burden on MCI in middle-aged and elderly Chinese. The guidelines of Strengthening the Reporting of Observational Studies in Epidemiology were followed in this study. All participants were recruited from the Department of Neurology, Beijing Chaoyang Hospital, Capital Medical University, from September 2018 to December 2020. We finally recruited 84 MCI patients according to the standard clinical and neuropsychological assessments. The diagnosis of MCI was made using the criteria published previously (Albert et al., 2011): (1) complaint of a change in cognition; (2) abnormal cognitive function in one or more domains; (3) independent activities in daily life; and (4) no dementia with Clinical Dementia Rating Scale < 1. Inclusion criteria were that patients were 45 years or older, underwent brain MRI scans within 1 month before the diagnosis of MCI, and agreed to participate in our study. Exclusion criteria included (1) patients with acute cerebrovascular diseases, neurodegenerative disease, and severe brain injury caused by toxins, infection, or inflammation; (2) the history of large-vessel infarction, cerebral hemorrhage, serious organ failure, systemic inflammatory disease, or cancer; (3) prominent visual or hearing impairment interfering with cognitive tests; (4) psychiatric disease or current use of medicines affecting cognitive function; and (5) subjects with poor-quality brain MRI or incomplete cognition test data.

Besides, 186 cognitively normal subjects for health and physical examination were recruited to control group at the same period. They underwent similar evaluation as MCI patients including clinical assessment, brain MRI, and neuropsychological testing battery. Other inclusion and exclusion criteria were the same as those in the MCI group.

This study was conducted in compliance with the Declaration of Helsinki. The research protocol was approved by the Ethics Committee of Beijing Chaoyang Hospital (2018-Sci-305). All subjects were informed of the objectives of this study, and their consent to participate was obtained.

### Magnetic Resonance Imaging Acquisition

Magnetic resonance imaging was performed on a 3.0-T MRI scanner (Prisma; Siemens AG, Erlangen, Germany) in the department of radiology in our hospital. The standardized sequences included T1-weighted, T2-weighted, fluid-attenuated inversion recovery, diffusion-weighted imaging, and susceptibility-weighted imaging.

### Total MRI CSVD Burden Assessment

Imaging markers of CSVD were defined according to Standards for Reporting Vascular Changes on Neuroimaging criteria described previously (Wardlaw et al., 2013). Periventricular and deep WMH was graded using the Fazekas scale (range 0–3, respectively) (Fazekas et al., 1987). CMB was evaluated according to Microbleed Anatomical Rating Scale (Gregoire et al., 2009). The severity of perivascular spaces (PVS) in the basal ganglia (BG) and centrum semiovale (CSO) was divided into five grades (0 for no PVS, 1 for 1–9 PVS, 2 for 10–20 PVS, 3 for 21–40 PVS, and 4 for >40 PVS) (Maclullich et al., 2004). Brain atrophy was evaluated using the visual rating scale for posterior atrophy (PA), in which scores 0–3 represent absent, mild, moderate, and severe PA, respectively (Koedam et al., 2011). Besides, we count the total numbers of lacuna lesions and PVS in the hippocampus.

We used a scale to represent the total burden of CSVD by counting each neuroimaging feature (range 0–4) (Staals et al., 2014). One point was awarded for each of the following items: one or more lacunas, one or more CMBs, periventricular WMH Fazekas score of 3 and/or deep WMH Fazekas score ≥ 2, and PVS for grades 2–4 in BG.

All images were evaluated by two neurologists blinded to the clinical data. Interrater reliability tests were performed in 30 subjects for each CSVD marker assessment, and the κ coefficients were 0.757–0.896 indicating good reliability. Disagreement was resolved by discussing with other coauthors.

### Clinical Data Collection

General characteristics including age, sex, education, body mass index, present cigarette, or alcohol use, medication history, and history of hypertension, diabetes, hyperlipidemia, ischemic stroke, transient ischemic attack (TIA), and cardiovascular diseases were collected according to medical records. Laboratory examinations were performed before or after MRI scans within 7 days, including tests for glycosylated hemoglobin, high-density lipoprotein cholesterol, low-density lipoprotein cholesterol, and homocysteine.

### Neuropsychological Assessment

For the assessment of cognitive performance, all participants underwent a face-to-face neuropsychological test by two trained interviewers. This protocol was based on the Leukoaraiosis and Disability in the Elderly study involving two global cognitive functioning tests and eight second-level tests that covered three important cognitive domains (Madureira et al., 2006). The tests used in this study included Mini-Mental State Examination (MMSE), Montreal Cognitive Assessment (MoCA), World Health Organization University of California-Los Angeles Auditory Verbal Learning Test (including Immediate Recall, Delayed Recall, and Recognition), Digit Span Test (including forward and backward), Trail-Making Test (TMT, including Part A and Part B), Stroop Test (including Parts A, B, and C), Verbal Fluency Test (animal), Digit-Symbol Substitution Test, Maze, and Digit Cancelation Test. The memory-domain score is a compound score of the mean of z-scores from Immediate Recall, Delayed Recall, Recognition (hits minus false positives), and Digit Span (backward). The executive function was calculated using the mean of z-scores of Stroop-C minus Stroop-B, TMT-B minus TMT-A, Digit-Symbol Substitution Test, and Verbal Fluency Test. Speed and motor control was evaluated as the mean of the z-scores from TMT-A, Digit Cancelation, and Maze. Z-scores of tests were inverted to (-z) if the higher scores represented worse performances.

### Calculation of Sample Size

This is a 1:2 unmatched case–control study. According to the available literatures, the prevalence of CSVD in MCI patients and controls was 72.7 and 47.1%, respectively (Jiang et al., 2019; Wong et al., 2021). The required sample size was calculated with a required power of 0.90 and an alpha of 0.05, resulting in the highest sample size for cases (*n* = 59). Assuming a loss-to-follow-up rate of 15%, recruitment numbers were set to 68 for cases and 136 for controls.

### Statistical Analysis

The continuous data following normal distribution were presented as mean and standard deviation, and independent sample t-test or one-way analysis of variance was used to determine significant differences among groups. Continuous data with non-normal distribution and ordinal variables were presented as median with quartiles and compared using the Mann–Whitney *U* test or Kruskal–Wallis test. Absolute numbers and percentages were presented for categorical variables, and the chi-square test was used to compare the differences between groups.

As for the PSM, a 1:2 nearest-neighbor matching approach without replacement was employed with a caliper of 0.15. Firstly, a binary logistic regression model was built according to outcome variables (MCI/control group) with the forward stepwise method. Then variables (age, hyperlipidemia, and stroke/TIA) that entered into the model were selected as covariates for PSM. After matching, the relative multivariate imbalance L1 measure was 0.209, which was much smaller than it was before PSM (0.358). Moreover, the absolute values of the standardized differences were all less than 10%, so the balance between two groups after matching was considered to be good. Finally, 74 MCI patients and 129 controls were successfully matched and included in the subsequent analyses.

The correlation between total MRI CSVD burden and cognitive function was examined using Spearman correlation analysis. Binary logistic regression analysis was performed to determine whether the total MRI CSVD burden was an independent risk factor of MCI after adjusting for confounders. Multiple linear regression analysis was used to explore the relationship between total CSVD score and neuropsychological test results.

Statistical Product and Service Solutions (version 22.0) was used for these analyses, and the built-in PSM module was used for PSM approach. The difference was considered statistically significant when *p* < 0.05.

## Results

**FIGURE 1:**
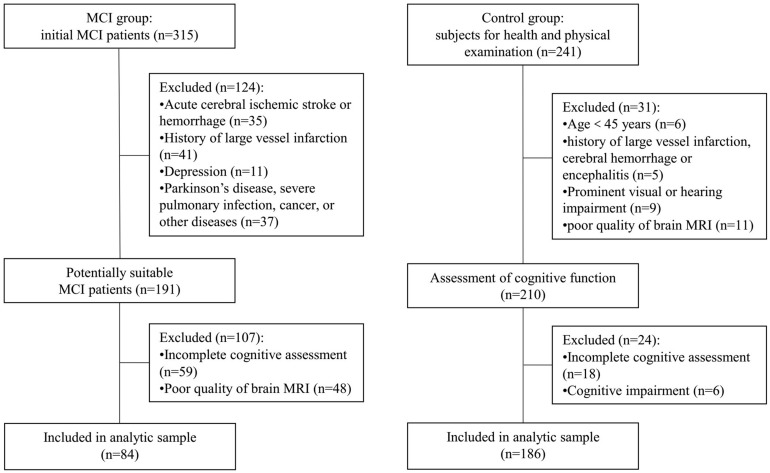
Flowchart of the subjects’ screening. MCI, mild cognitive impairment; MRI, magnetic resonance imaging.

Initially, there were 315 MCI patients registered in this study. According to the inclusion and exclusion criteria, 124 patients were excluded because of the acute cerebral ischemic stroke, cerebral hemorrhage, history of large-vessel infarction, and Parkinson’s disease, severe pulmonary infection, depression, or other diseases. Fifty-nine patients were excluded because they had visual or hearing impairment and did not finish all the cognitive tests. Besides, 48 patients’ brain MRI images were not clear enough to assess markers of CSVD. Finally, 84 MCI patients were included in the analyses. As for the subjects for health and physical examination in our hospital, a total of 186 cognitively normal individuals were included in the control group after excluding those who were younger than 45 years of age, had substandard MRI, did not complete the full cognitive assessment, and had some diseases required to be excluded. The flowchart is shown in Figure 1.

The general characteristics for the included subjects are presented in Table 1. Before PSM, there was no significant difference in sex ratio between two groups (male: 53.2 vs. 63.1%, *p* = 0.130). MCI patients were older (68.3 vs. 65.7 years, *p* = 0.025) and more with history of stroke or TIA (32.1 vs. 14.0%, *p* = 0.001) than the control group. After matching, the mean age was 67.9 years, and 63.5% of patients were male in the MCI group. For controls, the mean age was 67.2 years, and 55.0% of subjects were male. The PSM procedure was successful because it eliminated the between-group differences on all of the confounders (Table 1).

**TABLE 1:** Demographic data and clinical characteristics before and after propensity score matching.

	Before matching (*n* = 270)			After matching (*n* = 203)		
	Control group	MCI group	*p*	Control group	MCI group	*p*
*N*,%	186 (68.9)	84 (31.1)		129 (63.5)	74 (36.5)	
Age ^a^, years	65.7 (8.3)	68.3 (9.3)	0.025*	67.2 (8.0)	67.9 (9.2)	0.595
Men, *n* (%)	99 (53.2)	53 (63.1)	0.130	71 (55.0)	47 (63.5)	0.239
Years of education^b^, years	9 (9, 12)	9 (8, 12)	0.054	9 (9, 12)	9 (8, 12)	0.160
Cardiovascular risk factors/diseases						
BMI^a^, kg/m^2^	25.6 (3.6)	24.9 (3.6)	0.116	25.6 (3.7)	25.0 (3.6)	0.280
Smoking, *n* (%)	74 (39.8)	32 (38.1)	0.792	52 (40.3)	28 (37.8)	0.729
Drinking, *n* (%)	49 (26.3)	26 (31.0)	0.434	31 (24.0)	23 (31.1)	0.274
Hypertension, *n* (%)	122 (65.6)	52 (61.9)	0.558	86 (66.7)	45 (60.8)	0.401
Diabetes mellitus, *n* (%)	52 (28.0)	33 (39.3)	0.064	35 (27.1)	28 (37.8)	0.113
Cardiovascular diseases, *n* (%)	40 (21.5)	11 (13.1)	0.102	27 (20.9)	10 (13.5)	0.188
History of stroke/TIA, *n* (%)	26 (14.0)	27 (32.1)	0.001*	24 (18.6)	18 (24.3)	0.333
History of hyperlipidemia, *n* (%)	57 (30.6)	17 (20.2)	0.076	34 (26.4)	17 (23.0)	0.593
Medication use,% (*n*)						
Blood pressure-lowering medication	109 (58.6)	43 (51.2)	0.256	75 (58.1)	37 (50.0)	0.262
Salicylate/anticoagulant	42 (22.6)	23 (27.4)	0.393	31 (24.0)	18 (24.3)	0.963
Statin	48 (25.8)	14 (16.7)	0.098	32 (24.8)	12 (16.2)	0.153
Laboratory results						
HDL-c^b^, mmol/L	1.1 (0.9, 1.3)	1.1 (0.9, 1.3)	0.209	1.1 (0.9, 1.3)	1.1 (0.9, 1.3)	0.400
LDL-c^a^, mmol/L	2.7 (0.9)	2.5 (0.8)	0.302	2.6 (0.8)	2.6 (0.8)	0.832
HbA1C^b^, %	6.0 (5.7, 6.7)	6.0 (5.6, 7.0)	0.879	6.0 (5.7, 6.7)	6.0 (5.6, 7.0)	0.503
Homocysteine^b^, μmol/L	14.0 (11.0, 16.3)	14.0 (12.0, 17.0)	0.456	14.0 (11.0, 18.0)	14.0 (12.0, 17.0)	0.564
Brain MRI markers						
Presence of lacuna, *n* (%)	64 (34.4)	41 (48.8)	0.025*	45 (34.9)	33 (44.6)	0.171
Presence of CMB, *n* (%)	57 (30.6)	48 (57.1)	<0.001*	43 (33.3)	39 (52.7)	0.007*
3 periventricular WMH, *n* (%)	17 (9.1)	17 (20.2)	0.011*	14 (10.9)	14 (18.9)	0.109
2–3 deep WMH, *n* (%)	55 (29.6)	42 (50.0)	0.001*	41 (31.8)	36 (48.6)	0.017*
2–4 BG-PVS, *n* (%)	56 (30.1)	35 (41.7)	0.063	40 (31.0)	31 (41.9)	0.118
2–4 CSO-PVS, *n* (%)	121 (65.1)	57 (67.9)	0.653	89 (69.0)	51 (68.9)	0.991
Number of PVS in the hippocampus^b^	3 (2, 6)	3 (1, 4)	0.033*	3 (2, 6)	3 (1, 4)	0.048*
Presence of PA, *n* (%)	88 (47.3)	46 (54.8)	0.257	69 (53.5)	40 (54.1)	0.938
Total CSVD score^b^	1 (0, 2)	2 (1, 3)	<0.001*	1 (0, 2)	2 (1, 3)	0.006*

MCI, mild cognitive impairment; BMI, body mass index; TIA, transient ischemic attack; HDL-c, high-density lipoprotein cholesterol; LDL-c, low-density lipoprotein cholesterol; HbA1C, glycosylated hemoglobin; MRI, magnetic resonance imaging; CMB, cerebral microbleed; WMH, white matter hyperintensity; BG, basal ganglia; CSO, centrum semiovale; PVS, perivascular spaces; PA, posterior atrophy; CSVD, cerebral small vessel disease. ^a^Mean (standard deviation). ^b^Median (quartiles). *p < 0.05.

**FIGURE 2:**
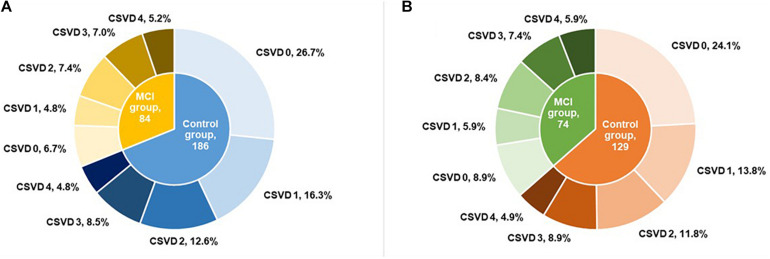
Proportion for different categories of total MRI CSVD burden before **(A)** and after **(B)** propensity score matching. MRI, magnetic resonance imaging; CSVD, cerebral small vessel disease; MCI, mild cognitive impairment.

Before PSM, 90 (33.3%) patients had no marker of CSVD, 57 (21.1%) had one score, 54 (20.0%) had two scores, 42 (15.6%) had three scores, and 27 (10.0%) had four scores of total CSVD burden. After PSM, there were 67 (33.0%) patients with no marker of CSVD, 40 (19.7%) with one score, 41 (20.2%) with two scores, 33 (16.3%) with three scores, and 22 (10.8%) with four scores of total CSVD burden. The proportions for different categories of total MRI CSVD burden in the MCI group and control group are shown in Figure 2. Compared with controls, the MCI group had more severe CSVD burden (Table 1) and showed poorer performances in global cognitive function and three important cognitive domains with *p* < 0.001 (Table 2).

**TABLE 2:** Comparison of cognitive function between two cognition groups after propensity score matching.

	Control group	MCI group	*p*
MMSE^b^	29 (29, 30)	25 (23, 27)	<0.001*
MoCA^b^	27 (26, 28)	22 (19, 24)	<0.001*
Memory^a^	0.28 (0.59)	–0.59 (0.83)	<0.001*
Executive function^a^	0.24 (0.45)	–0.55 (0.72)	<0.001*
Speed and motor control^a^	0.19 (0.65)	–0.53 (1.01)	<0.001*

MCI, mild cognitive impairment; MMSE, Mini-Mental State Examination; MoCA, Montreal Cognitive Assessment. ^a^Mean (standard deviation). ^b^Median (quartiles). *p < 0.05.

We firstly investigated the role of total MRI CSVD burden in MCI patients and people with normal cognition (Table 3). In the binary logistic regression analysis, the score of CSVD burden was associated with MCI independent of age, sex, and years of education (Model 1: *p* = 0.019). Brain atrophy and PVS in CSO and hippocampus were not included in the total CSVD burden scale used in this study. So, we added them to the regression analysis in order to adjust for their potential effects on cognitive function, and the above result remained stable (Model 2: *p* = 0.014).

**TABLE 3:** Binary logistic regression analysis for the association between total CSVD burden and MCI after propensity score matching.

	*B*	*S.E.*	*Wald*	*OR*	95% *CI*	*p*
Model 1	0.26	0.11	5.46	1.30	1.04, 1.62	0.019*
Model 2	0.28	0.12	6.10	1.33	1.06, 1.67	0.014*

CSVD, cerebral small vessel disease; MCI, mild cognitive impairment; S.E., standard error; OR, odds ratio; 95% CI, 95% confidence interval. Model 1, adjusted for age, sex, and years of education. Model 2, model 1 + brain atrophy, perivascular space in the hippocampus and perivascular space in the centrum semiovale. *p < 0.05.

Then, we compared the cognitive function among different categories of CSVD burden (Table 4). There were significant differences among five grades of CSVD burden in scores of MMSE (*p* = 0.048), MoCA (*p* = 0.004), memory (*p* = 0.009), executive function (*p* < 0.001), and speed and motor control (*p* = 0.006). Spearman correlation analysis showed that total MRI CSVD burden was positively correlated with MCI (*rho* = 0.193, *p* = 0.006).

**TABLE 4:** Comparison for cognitive function among different grades of total MRI CSVD burden after propensity score matching.

	Total MRI CSVD burden					*p*
	0 (*n* = 67)	1 (*n* = 40)	2 (*n* = 41)	3 (*n* = 33)	4 (*n* = 22)	
MMSE^b^	29 (27, 30)	29 (27, 29)	29 (26, 30)	27 (25, 30)	26 (22, 29)	0.048*
MoCA^b^	26 (24, 28)	25 (23, 27)	26 (23, 27)	25 (22, 28)	23 (19, 26)	0.004*
Memory^a^	0.25 (0.75)	–0.09 (0.63)	–0.18 (0.84)	–0.21 (0.89)	–0.28 (0.89)	0.009*
Executive function^a^	0.19 (0.61)	0.07 (0.56)	–0.13 (0.80)	–0.25 (0.57)	–0.54 (0.65)	<0.001*
Speed and motor control^a^	0.15 (0.90)	0.08 (0.61)	–0.18 (0.91)	–0.27 (0.90)	–0.53 (0.84)	0.006*

MRI, magnetic resonance imaging; CSVD, cerebral small vessel disease; MMSE, Mini-Mental State Examination; MoCA, Montreal Cognitive Assessment. ^a^Mean (standard deviation). ^b^Median (quartiles). *p < 0.05.

We investigated the relationship between scores of total MRI CSVD burden and performances in different cognitive domains. Spearman correlation analysis indicated that scores of cognitive tests were negatively correlated with severity of total CSVD burden (Table 5). Multivariate linear regression analyses showed that higher total MRI CSVD burden was associated with the deficit of cognitive performance in global cognition and each cognitive domain such as memory, executive function, and speed and motor control after adjusting for age, sex, and education (Table 6, Model 1). Moreover, PA, CSO-PVS, and PVS in the hippocampus were added into analyses as extra confounders (Table 6, Model 2). The results also showed that total MRI CSVD burden was an independent risk factor of cognitive dysfunction in all cognitive domains.

**TABLE 5:** Spearman correlation analysis of total CSVD burden and cognitive function after propensity score matching.

	MCI	MMSE	MoCA	Memory	Executive function	Speed and motor control
*rho*	0.193	–0.205	–0.256	–0.232	-0.382	–0.311
*p*	0.006*	0.003*	<0.001*	0.001*	<0.001*	<0.001*

CSVD, cerebral small vessel disease; MCI, mild cognitive impairment; MMSE, Mini-Mental State Examination; MoCA, Montreal Cognitive Assessment. *p < 0.05.

**TABLE 6:** Association between total CSVD burden and cognitive domains after propensity score matching.

	MMSE		MoCA		Memory		Executive function		Speed and motor control	
	β (95% *CI*)	*p*	β (95% *CI*)	*p*	β (95% *CI*)	*p*	β (95% *CI*)	*p*	β (95% *CI*)	*p*
Model 1	–0.21 (–0.90, –0.20)	0.002*	–0.19 (–0.99, –0.19)	0.004*	–0.16 (–0.17, –0.01)	0.021*	–0.28 (–0.20, –0.08)	<0.001*	–0.18 (–0.19, –0.04)	0.004*
Model 2	–0.21 (–0.91, –0.21)	0.002*	–0.20 (–1.00, –0.19)	0.004*	–0.17 (–0.18, –0.02)	0.012*	–0.28 (–0.20, –0.08)	<0.001*	–0.19 (–0.20, –0.04)	0.004*

CSVD, cerebral small vessel disease; MMSE, Mini-Mental State Examination; MoCA, Montreal Cognitive Assessment; 95% CI, 95% confidence interval. Model 1, adjusted for age, sex, and years of education. Model 2, model 1 + brain atrophy, perivascular space in the hippocampus and perivascular space in the centrum semiovale. *p < 0.05.

## Discussion

In this case–control study, we reported that higher total CSVD burden played an important role in cognitive disorder. The majority of MCI patients had at least one identifiable CSVD marker. Compared with controls, the MCI group had more severe CSVD burden. After PSM, binary logistic regression analysis indicated that the total MRI CSVD burden was an independent risk factor of MCI. There were significant differences among five grades of CSVD burden on global cognition (MMSE and MoCA) and important cognitive domains (memory, executive function, and speed and motor control). Multivariate linear regression analyses showed that higher total MRI CSVD burden was associated with the deficit of cognitive performance in global cognition and above three cognitive domains.

A population-based study showed that the prevalence of any one CSVD was 47.1% in Han Chinese aged 55–65 years (Jiang et al., 2019). According to our small sample data, the prevalence (before and after PSM: 78.6 and 75.7%) of CSVD and the mean total CSVD score (before and after PSM: 2.0 and 1.9) of MCI patients were similar with those reported in the previous cohort (Banerjee et al., 2018; de Heus et al., 2020; Wong et al., 2021). These results further confirmed their inference that the burden of CSVD in cognitive disorder patients was higher than that in healthy people and populations with other diseases (TIA, lacunar infarction, and hypertension) (Banerjee et al., 2018).

Previous studies mostly focused on the pathological mechanism and clinical manifestation of single imaging markers of CSVD, such as WMH, CMB, and lacunar infarction. However, alone with the recent proposal of CSVD as a whole-brain disease, the harm from the total CSVD burden to the cognitive function has become a hot topic in this field. A study based on patients with first-ever acute ischemic stroke showed that CSVD burden was associated with decreased MMSE score (Liang et al., 2019). The results of the Leukoaraiosis and Disability Study indicated that a combined measure of CSVD strongly predicted cognitive and functional outcomes even above the contribution of individual markers (Jokinen et al., 2020). A Polish observational study suggested that MCI and CSVD were highly prevalent in the middle-aged population, and severe CSVD was related to twofold incidence of MCI (Szcześniak et al., 2020). Our results also indicated that the total CSVD MRI burden was an independent risk factor of MCI, which was consistent with most previous studies. However, another cross-sectional study came to the opposite conclusion in which the total CSVD score was not significantly correlated with cognition in post-stroke CSVD patients, while structural brain network measures were more useful in predicting early cognitive impairment (Du et al., 2019). Since there is controversy, more causal researches with a larger population are necessary in the future.

As for performances in different cognitive domains, our study showed that higher total burden of CSVD was associated with overall cognitive impairment, which suggested a non-specific neuropsychological profile for CSVD patients. An early study suggested that multiple cognitive domains were impaired in the MCI-CSVD group, while MCI-prodromal Alzheimer’s disease patients demonstrated greater memory impairment with relatively preserved mental processing speed function (Zhou and Jia, 2009). In a memory clinic population, the total CSVD score was associated with performances of frontal and visuospatial tasks (Banerjee et al., 2018). Also, for the post-stroke cognitive decline, total CSVD burden was specifically related with decreased MMSE subscores including orientation, calculation, and word recall (Liang et al., 2019). These different results may be due to those heterogeneities in study populations, study designs, cognitive measurement methods, sample sizes, and so on. More well-designed studies with multidimensional neuropsychiatric assessments will be needed to explore the cognitive feature of CSVD patients. Tests tapping these characteristic domains might be potentially useful for the early detection of cognitive impairment patients caused by different diseases and choosing targeted treatment methods.

When multiple markers of CSVD are present at the same time, they may have synergistic or superimposed effects on cognitive dysfunction (Jokinen et al., 2012; Ye et al., 2019). The underlying mechanism of CSVD leading to cognitive impairment is still under investigation. CSVD lesions may lead to the direct damage of surrounding brain tissues, causing the debilitation on axonal communication or the disconnection of cortical and subcortical tracts (Charidimou and Werring, 2012; Coban et al., 2017). Furthermore, CSVD are more likely to imply more remote and generalized effects. CSVD can disrupt the integrity of brain structural and functional networks, thereby leading to cognitive impairment. Finally, other neurodegenerative pathologies can be present at the same time and are also involved in the pathogenesis of cognitive dysfunction (Ter Telgte et al., 2018).

This study was designed as a case–control study in order to provide more evidence for the inference of causality. The main strength of our study includes the use of various neuropsychological tests and composite z-scores to improve the sensitivity of detecting cognitive decline and limit the false statistical differences. Another strength is that the PSM approach makes the comparison between groups more suitable by addressing the covariate imbalance, so as to minimize the selection bias in observational studies. This useful analysis would help to provide more cogent evidence on the impact of CSVD on MCI and better understand the pathophysiological process for dementia. However, there are also some limitations in our study. Firstly, this is a retrospective single-center study, which may lead to selection and information bias. Secondly, the severity of CSVD was assessed only using semiquantitative scales. Although these scales were widely used for the assessment of total MRI CSVD burden in a simple and pragmatic way, there were still some shortages. Advanced software applications used to segment structures and make quantitative analysis for brain MRI might be employed in the further study to provide more accurate information and more reliable conclusions. Lastly, because of the relatively small sample size, subtypes of MCI were not further classified and analyzed in our study. The present study examined the overall impact of CSVD on cognitive impairment.

In summary, the results after PSM supported that the total MRI CSVD burden was an independent risk factor of MCI. Moreover, higher CSVD burden was associated with the overall cognitive impairment among middle-aged and elderly people in China. There is clearly a need for prospective, multicenter studies using advanced imaging technology and post-processing software to aid in clinical decision-making regarding the diagnosis and treatment of CSVD and cognitive impairment in the future.

## Data Availability Statement

The raw data supporting the conclusions of this article will be made available by the authors, without undue reservation.

## Ethics Statement

The studies involving human participants were reviewed and approved by the Ethics Committee of Beijing Chaoyang Hospital, China. The patients/participants provided their written informed consent to participate in this study.

## Author Contributions

WH and JY: contributed to the conception and design of the research. XL, JY, SY, and LY: collected the data. XL and SY: contributed to the analysis and interpretation of the data. XL: wrote the first draft of the manuscript. WQ, LY, YL, and WH: helped with the critical revision of the manuscript. All authors contributed to the article and approved the submitted version.

## Conflict of Interest

The authors declare that the research was conducted in the absence of any commercial or financial relationships that could be construed as a potential conflict of interest. The reviewer YH declared a shared affiliation, with no collaboration, with several of the authors XL, WQ, LY, SW, YL, and WH to the handling editor at the time of the review.
